# Time- and Temperature-Dependent Effects of PHBV on Physiological Responses in Brine Shrimp

**DOI:** 10.3390/toxics14060533

**Published:** 2026-06-20

**Authors:** Natalia S. Buzzi, Anna Jáuregui, Anna Marín, Juan C. Navarro, Mar Llorca, Myriam Lizanda, María Constanza Díaz Andrade, Ana Carolina Moya, José Gámez-Pérez, Luis Cabedo, Inmaculada Varó

**Affiliations:** 1 Instituto Argentino de Oceanografía (IADO-CONICET/UNS), CCT-Bahía Blanca, Camino La Carrindanga, km 7.5, Edificio E1, Bahía Blanca B8000FWB, Buenos Aires, Argentina; 2 Departamento de Biología, Bioquímica y Farmacia, Universidad Nacional del Sur (UNS), San Juan 671, Bahía Blanca B8000ICN, Buenos Aires, Argentina; mcandrade@criba.edu.ar (M.C.D.A.); acmoya83@gmail.com (A.C.M.); 3 Grupo de Polímeros y Materiales Avanzados (PIMA), Universitat Jaume I, Avinguda de Vicent Sos Baynat, 12006 Castelló de la Plana, Catellón, Spain; ajauregu@uji.es (A.J.); anmarin@uji.es (A.M.); gamez@uji.es (J.G.-P.); lcabedo@uji.es (L.C.); 4 Instituto de Acuicultura Torre de la Sal (IATS, CSIC), 12595 Ribera de Cabanes, Castellón, Spain; jc.navarro@csic.es (J.C.N.); mllorcallovet@gmail.com (M.L.); myriamlizandapiqueras@gmail.com (M.L.); 5 Instituto de Ciencias Biológicas y Biomédicas del Sur (INBIOSUR), Universidad Nacional del Sur (UNS)-CONICET, San Juan 671, Bahía Blanca B8000ICN, Buenos Aires, Argentina; 6 Grupo de Investigación en Morfología e Histología Animal Comparada (GIMHAC) (INBIOSUR-UNS-CONICET), Bahía Blanca B8000ICN, Buenos Aires, Argentina

**Keywords:** microplastics, bioplastics, PHBV, brine shrimp, biomarkers, fatty acids, temperature

## Abstract

Aquatic organisms are exposed to multiple stressors, including microplastic pollution and rising temperatures. Bioplastics like Poly(3-hydroxybutyrate-co-3-hydroxyvalerate) (PHBV) are considered sustainable alternatives to conventional plastics, although their biological effects remain poorly understood. This study evaluated the effects of PHBV microplastics on *Artemia franciscana* under different temperature and exposure conditions. Organisms were exposed to 25 and 100 mg·L^−1^ PHBV for 7, 14, and 21 days at 25 °C and for 14 days at 29 °C. Growth, development, antioxidant enzyme (CAT, GST) and esterase activities (ChE, CbE), lipid peroxidation (LPO), gut histology, fatty acid profiles and polymer particle length distributions were assessed. Growth and development increased with PHBV concentration, exposure time, and temperature. Enzymatic activities and LPO were significantly affected by these factors, although no evidence of oxidative damage was detected. Marked gut lesions were observed at 100 mg·L^−1^ PHBV at 29 °C after 14 days. Fatty acid profiles were mainly influenced by time and temperature, while high PHBV levels were associated with additional, more subtle changes in long-chain polyunsaturated fatty acids. PHBV particle length distributions also varied depending on exposure conditions. These findings suggest that PHBV induces physiological responses distinct from those typically reported for conventional microplastics and highlight the importance of considering multiple stressors in ecotoxicological studies.

## 1. Introduction

Bioplastics, many of which are based on biopolymers, comprise a group of plastic materials that are produced entirely or partially from renewable sources (“bio-based”), including microorganisms, plants, and waste materials, and provide a sustainable alternative to reduce petroleum-based plastic production and accumulation in the environment [1]. Additional advantages include their lower associated carbon emissions and energy requirements during production, as well as their faster biodegradation into harmless compounds such as H_2_O, CO_2_, biomass and minerals [2,3]. Polyhydroxyalkanoates (PHAs) are one of the main commercially available bioplastics. Among them, Poly(3-hydroxybutyrate-*co*-3-hydroxyvalerate (PHBV) is a PHA copolymer of increasing interest as a potential substitute for conventional plastics due to its mechanical properties comparable to petroleum-based polymers [4]. Currently, the substantial increase in bioplastic production and consumption, projected to reach 6.3 million tonnes by 2027, has raised critical questions regarding their environmental implications [5]. Despite being marketed as sustainable, their incomplete degradation in natural conditions may potentially lead to long-term accumulation comparable to that reported for some conventional synthetic plastics [6] and the release of harmful chemical additives [7].

As with conventional plastics, incomplete degradation of bioplastics in aquatic environments might generate tiny particles, often becoming microplastics (MPs < 5 mm) [8,9]. The widespread occurrence of MPs in aquatic ecosystems has emerged as a major global environmental concern, with concentrations ranging from µg·L^−1^ to a few mg·L^−1^, with higher values reported in heavily contaminated or localized hotspot areas [10,11]. However, equivalent data for micro-bioplastics remain largely unknown owing to their lower prevalence when environmental samples are analyzed [12,13]. Evidence of MP presence across diverse freshwater and marine organisms [14,15] indicates biological impacts, as previous studies have documented detrimental effects of conventional MPs, including tissue damage, behavioral and growth alterations, nutritional deficiencies, and impaired reproduction [16,17,18]. At the cellular level, MPs may alter metabolic processes and cause oxidative stress (e.g., [19,20]), which may consequently lead to metabolic dysregulation, neurotoxicity, and cytotoxicity [21].

Given that MPs can be easily transferred to higher trophic levels via ingestion [22], evaluating their effects on lower-trophic organisms like zooplankton is essential since these organisms are relevant food sources and play a central role in biomagnification within the food web [23]. In this sense, Jaapar et al. [24] observed that biodegradable PHA microbeads were less harmful to the copepod *Nitokra lacustris pacifica* than synthetic polystyrene (PS) microbeads. PS caused a significant reduction in algal ingestion and negatively affected reproductive success, development, and survival rate in the copepods. Di Giannantonio et al. [25] found that neither polylactic acid (PLA) nor synthetic polyvinylidene fluoride (PVDF) affected the survival of the microcrustacean *Artemia franciscana* and the cnidarian *Aurelia* sp. (common jellyfish), but both MPs altered the pulsation mode of the jellyfish. Conversely, Charoeythornkhajhornchai et al. [26] demonstrated that two biodegradable bioplastics, PLA and polybutylene succinate (PBS), and synthetic polyethylene (PE) particles reduced the hatching percentage and increased the mortality of *A. franciscana*, in both cases dependent on the concentration of particles and the exposure time. Recently, Afifah et al. [6] showed that the growth and survival of *A. franciscana* were not altered by PHA, PLA, or PBS. Therefore, while bioplastics offer environmental advantages, their ecological consequences require thorough evaluation. Moreover, the effects of plastic particles derived from both conventional plastics and bioplastics on aquatic organisms may be influenced by climate change-related stressors, particularly rising water temperatures, highlighting the need to assess their impacts under future environmental scenarios.

Global warming, as a primary driver of climate change, is accelerating the increase in water temperatures, resulting in substantial impacts on marine ecosystems. The Intergovernmental Panel on Climate Change (IPCC) has predicted an increase of up to 4 °C in ocean surface temperatures by the year 2100 [27]. Temperature increase not only affects the physiology, growth, and survival of aquatic organisms [28,29] but also raises the toxicity of many pollutants, which is known as the “climate-induced toxicant sensitivity” (CITS) concept [30,31]. Most studies related to the impacts of global warming on the effects of pollutants have focused on traditional pollutants such as metals and pesticides [32,33]. However, attention has shifted recently to the effects on MPs. The few studies on this topic show that high temperatures exacerbate the negative impact of conventional MPs on aquatic ecosystems [34,35,36,37]. Furthermore, warming conditions may alter MP toxicity, leading to synergistic interactions that enhance behavioral and physiological responses in invertebrates [35,38]. Han et al. [35] showed that PS MPs and temperature have a combined long-term adverse effect on the survival and growth of *A. franciscana* after long-term exposure, especially at relatively high temperatures (30 °C) and MP concentrations (2 mg·L^−1^). Moreover, PS induced oxidative and immunological stress under the conditions mentioned. Despite growing interest in bioplastics, the available literature addressing their long-term effects on marine invertebrates remains limited, particularly under combined environmental stressors such as temperature. A recent study performed with different types of bioplastic microparticles (PHA, PLA and PBS) showed that the growth of *A. franciscana* was significantly decreased at 30 °C compared to 26 °C, while survival was affected by bioplastic concentration, showing a higher survival percentage at 0.2 mg·L^−1^ than at 2 mg·L^−1^ [6]. Therefore, understanding the combined effects of elevated temperature and bioplastic exposure on marine organisms, specifically on zooplankton, is crucial for assessing the potential ecological risks associated with both stressors.

Within the zooplankton, *Artemia*, commonly known as brine shrimp, is a microcrustacean widely used as a model organism in ecotoxicological studies, particularly for assessing the effects of plastic particles on marine biota [39,40]. In previous studies, we evaluated the effects of PHBV exposure on *A. franciscana*. We demonstrated that long-term exposure to PHBV did not affect *A. franciscana* survival, promoted its growth, and reduced development time without altering feeding behavior. However, it caused adverse impacts on intestinal tissues and changes in fatty acid composition, suggesting limited physiological disruption compared to conventional MPs [41].

Although *A. franciscana* is highly thermotolerant and capable of adapting to a wide range of thermal regimes, optimal survival, growth, and reproductive performance are typically reported between 20 and 28 °C under laboratory conditions [42,43]. To further elucidate the effects of PHBV on *A. franciscana*, this study investigates the influence of PHBV concentration and temperature on biological responses. Accordingly, to assess the combined effects of temperature and bioplastic exposure on *Artemia* performance, the temperatures selected here (25 and 29 °C) encompass the optimal range and a slightly elevated condition, with the upper temperature representing a moderate increase consistent with projected future warming scenarios.

In this context, our hypothesis is that exposure to elevated PHBV microplastic concentrations, exceeding currently reported environmental levels, would induce biological alterations in *A. franciscana* and that these responses would vary according to exposure time, PHBV concentration, and temperature. To test this hypothesis, this study aimed to evaluate the effects of exposure to PHBV microplastics at concentrations representing high-exposure scenarios on the growth, development, biochemical changes, and digestive tract integrity of *A. franciscana* under different exposure durations. Furthermore, we investigated whether high temperature (29 °C), as a climate change-related stressor, could modulate the biological responses associated with PHBV exposure.

## 2. Materials and Methods

### 2.1. Bioplastic Particle Preparation

PHBV powder (commercial grade ENMAT Y1000P, containing 3 wt.% 3-hydroxyvalerate, with a density of 1.23 g/cm^3^) was purchased from Tianan Biologic Material Co., Ltd. (Ningbo, China). The chemical structure of PHBV is shown in Figure S1. An MP suspension (500 mg·L^−1^) was prepared using natural seawater (NSW, 36 psu), which had been filtered through a 0.2 μm Whatman mixed cellulose ester filter. The suspension was sonicated before use and then diluted with filtered NSW as needed.

### 2.2. Hatching and Experimental Design

Dried cysts of *A. franciscana* used in the experiments were purchased at INVE Aquaculture (Dendermonde, Belgium). Instar I nauplii were obtained following Varó et al. [44] by incubating the cysts for 22 h in filtered natural seawater (NSW, 5 μm polypropylene filter/Atlas Filtri, Limena, Italy) at 28 °C under constant light and aeration. After hatching, the nauplii were separated from the empty shells and any unhatched cysts, then transferred to glass beakers containing filtered NSW at a density of 0.5 nauplii·mL^−1^. They were acclimated for 24 h in a thermostatic chamber (25 ± 0.5 °C or 29 ± 0.5 °C, 14:10 h light/dark cycle) without feeding until they reached the Instar II–III stage.

Experiment 1 (Exp. 1): Instar II–III nauplii (0.5 individuals·mL^−1^) were exposed under controlled conditions (25 ± 0.5 °C; 14:10 h light/dark cycle) and moderate aeration for 21 days to two PHBV concentrations (25 and 100 mg·L^−1^, PHBV25 and PHBV100, respectively). Fresh microalgae (*Tetraselmis suecica*) were supplied at a density of 2 × 10^5^ cells·mL^−1^ during the first 3 days, subsequently increased to 4 × 10^5^ cells·mL^−1^ for the remainder of the experiment. A control treatment without PHBV (CTRL; 0 mg·L^−1^) with microalgae was included. Five independent replicates were set for each concentration. To maintain consistent exposure conditions and minimize fungal and bacterial contamination, the medium was fully renewed twice per week. After 7, 14 and 21 days (end of the assay), organisms were randomly sampled from each condition to analyze the parameters described below.

Experiment 2 (Exp. 2): To study the effect of temperature, Instar II–III *A. franciscana* were exposed to PHBV under the same conditions described above, but at 29 °C (29 ± 0.5 °C; 14:10 h light/dark cycle) for 14 days. After the exposure period, organisms were randomly sampled from each concentration to analyze the different parameters.

As in Buzzi et al. [41], PHBV concentrations several orders of magnitude higher than current environmental levels of MP contamination were used in the present study to elicit detectable sublethal and mechanistic responses under controlled laboratory conditions, mimicking worst-case scenarios such as hotspot conditions, pulse pollution events, and potential future increases in environmental concentrations [10,11]. These experimental levels therefore require cautious extrapolation to natural ecosystems.

### 2.3. Growth and Development

Growth and development stages were recorded at the beginning and on days 7, 14 and 21 in Exp. 1; and at the beginning and after 14 days of exposure in Exp. 2. The measurements obtained on day 14 in Exp. 1 were previously reported [41] and are included here as part of the same experimental framework to enable comparison across time points. A total of 25 organisms from each concentration (CTRL, PHBV25 and PHBV100) were randomly selected and photographed with a Leica MZ6 stereomicroscope (Leica Microsystems, Wetzlar, Germany) equipped with a Leica hz6 digital camera via Leica Application Suite (LAS v4.12) software. Growth and development were evaluated through the observation of the images using ImageJ software (1.54p) (National Institutes of Health, Bethesda, MD, USA). The organisms were measured from the top of the head to the furca.

### 2.4. Scanning Electron Microscopy (SEM) 

The surface morphology of the PHBV polymer was examined using a JEOL 7001F (JEOL Ltd., Tokyo, Japan) high-resolution field-emission scanning electron microscope operated at 5 kV. Prior to imaging, samples were sputter-coated with platinum for 15 s in an argon atmosphere (Bal-Tec SCD 500, Leica Microsystems, Pfäffikon, Switzerland).

As a baseline (day 0) condition in Exp. 1, PHBV particle suspensions at both tested concentrations in artificial seawater were filtered (20 µm pore size) to recover the particles prior to exposure to *A. franciscana*. These filtered samples were directly used for SEM analysis to characterize the initial surface morphology of the polymer.

To explore potential morphological changes associated with metabolic utilization of PHBV by *A. franciscana*, changes in the surface morphology of PHBV particles recovered from fecal pellets were assessed using the same procedure. After exposure, the medium was filtered, and the retained fecal pellets were collected for subsequent analysis. The analyzed samples corresponded to Exp. 1 (days 14 and 21) and Exp. 2 (day 14), as described in the experimental design section.

SEM images of PHBV particles were processed using ImageJ software to determine particle length distributions. Particle length was measured using the ‘Measure’ function, analyzing at least 200 randomly selected particles per condition. Measurements were calibrated using the SEM scale bar. Particle length distributions were explored using frequency histograms to visualize distribution patterns across conditions.

### 2.5. Biomarkers

One hundred organisms from each concentration (CTRL, 25, and 100 mg·L^−1^ PHBV) were selected for biochemical marker and lipid peroxidation analysis. In Exp. 1, pools of 20 organisms per replicate were collected on days 7, 14, and 21; in Exp. 2, samples were collected on day 14. Lipid peroxidation results at 14 days from Exp. 1 were previously reported [41]; here, they are included to allow comparison across time points within the same experimental framework. The organisms were rinsed with distilled water, weighed using an analytical balance, and immediately stored at −80 °C. The samples were processed according to Peixoto et al. [40], with slight modifications. Briefly, the organisms were manually homogenized at 4 °C in a glass homogenizer using a 1:3 wet-weight-to-buffer ratio in ice-cold phosphate buffer (100 mM, pH 7.4; 150 mM KCl, 1 mM EDTA, and 0.1% Triton X). Homogenates were centrifuged at 2348 *g* (4 °C) for 5 min, and two aliquots of 50 μL from the resulting supernatants were stored at −80 °C for lipid peroxidation analysis. The rest of the supernatants were centrifuged at 10,000 *g* (4 °C) for 10 min. Homogenate aliquots were stored at −80 °C for a maximum of 3 months prior to biomarker analysis to prevent enzyme degradation.

Catalase activity (CAT) was measured according to Aebi [45] by measuring H_2_O_2_ concentration decrease at an absorbance of 240 nm in UV microplates (Greiner UV-Star^®^) during 3 min.

Glutathione-S-transferase activity (GST) was determined following Habig et al. [46] using 1-chloro-2,4-dinitrobenzene (CDNB) as the substrate, and the absorbance was read at 340 nm during 5 min.

Cholinesterase (ChE) activity was measured following the modified Ellman method using acetylcholine (ATC) as the substrate, and the absorbance was read at 415 nm during 15 min [40].

Carboxylesterase activity (CbE) was analyzed according to Mastropaolo and Yourno [47] adapted to microplates, using p-nitrophenyl acetate (pNPA) as the substrate, and the absorbance was read at 405 nm during 5 min.

Lipid peroxidation (LPO) was quantified following Varó et al. [48], based on the detection of thiobarbituric acid reactive substances (TBARSs) and expressed as malondialdehyde (MDA) equivalents. In brief, duplicate 50 μL aliquots were incubated with 8.1% sodium dodecyl sulfate supplemented with 0.005% butylated hydroxytoluene (BHT), 20% acetic acid (pH 3.5), and 0.8% thiobarbituric acid and subsequently heated in boiling water for 60 min. After cooling, 80 μL of distilled water and 500 μL of n-butanol–pyridine (15:1, *v*/*v*) were added, vigorously mixed, and centrifuged at 10,000 *g* for 5 min. The organic phase was collected, and fluorescence was measured at 515/553 nm (EX/EM). MDA concentrations were determined using a calibration curve generated with 1,1,3,3-tetraethoxypropane as the standard.

### 2.6. Fatty Acid Analysis

Lipid extraction and fatty acid methyl ester (FAME) preparation were carried out using solvents of >99% purity (Merck KGaA, Darmstadt, Germany). FAMEs were obtained following Garrido et al. [49], adapted for reduced sample volumes, from pools of five organisms (*n* = 3 per concentration; CTRL, PHBV25 and PHBV100) collected after 7, 14 and 21 days of exposure in Exp. 1 and after 14 days of exposure in Exp. 2. The fatty acid profiles at 14 days in Exp. 1 were previously published [41] and are included here to enable a comprehensive temporal analysis and facilitate temperature comparisons at 14 days. Purification was performed by thin-layer chromatography on 20 × 20 cm silica gel G60 plates (Merck KGaA), and FAMEs were analyzed using a Thermo Trace GC Ultra gas chromatograph (Thermo Electron Corporation, Waltham, MA, USA) equipped with an on-column injector, a flame ionization detector (FID), and a TR-WAX silica capillary column (30 m × 0.25 mm × 0.25 μm; Teknokroma, Barcelona, Spain). Helium was used as the carrier gas under a thermal gradient program (50 °C to 220 °C). Peaks were integrated with Azur Datlys software (St Martin d’Hères, Isère, France), and compounds were identified by comparison with a commercial standard (Supelco 37-component FAME mix, Merck KGaA) and reference fish oils.

### 2.7. Histology

*A. franciscana* were collected for histopathological evaluation of the digestive tract after 14 and 21 days at 25 °C (Exp. 1) and after 14 days at 29 °C (Exp. 2). Individuals from the CTRL, PHBV25 and PHBV100 were analyzed. Histopathological analysis at 14 days in Exp. 1 was previously published [41] and is included here to enable a complete temporal analysis and facilitate temperature comparisons at 14 days. For each concentration, five organisms were fixed in 10% buffered formalin and processed using an alternative dehydration method based on N-butyl alcohol [41,50]. Samples were embedded in Technovit 7100 resin. Histological analysis was carried out on 2 μm sections stained with hematoxylin and eosin using a Nikon Eclipse E600 light microscope (Tokyo, Japan) equipped with a Euromex DC 20000i camera and Euromex ImageFocus Alpha vx64 software (Diuven, Netherlans). The cell damage in the digestive epithelium of *A. franciscana* from Exps. 1 and 2 was classified according to the following semi-quantitative scale: 0: normal; 1: mild (less than 25% of damage); 2: moderate (less than 50% of damage); 3: severe (less than 75% of damage), and 4: very severe (more than 75% of damage). The damage indicators used for this analysis were: presence of blebbing cells, epithelium cell disruption, detachment of the epithelium, presence of edematous enterocytes and cell hyperplasia. The evaluation of the damage level was based on estimates from two trained independent researchers, blinded to the samples being observed.

### 2.8. Statistical Analysis

Normality and homoscedasticity of data were tested with Levene and Kolmogorov–Smirnov tests, respectively. When required, data were log-transformed (natural logarithm) to improve compliance with these assumptions.

#### 2.8.1. Growth and Biochemical Markers

In Exp. 1, the effects of PHBV concentration (CTRL, PHBV25 and PHBV100) and exposure time (7, 14 and 21 days) on growth and biochemical markers were evaluated using a two-way nested ANOVA, including replicate flasks (*n* = 5) as a factor nested within concentration. Additionally, day 14 data from Exp. 1 and Exp. 2 were combined and analyzed using a two-way ANOVA with concentration (CTRL, PHBV25 and PHBV100) and temperature (25 °C and 29 °C) as fixed factors.

#### 2.8.2. PHBV Particles on Fecal Pellets

Particle length data were summarized using box-and-whisker and violin plots and analyzed using two independent two-way ANOVA models: (i) concentration (PHBV25 and PHBV100) × exposure time (0, 14, and 21 days) based on Exp. 1 and (ii) concentration × temperature (25 °C and 29 °C) at day 14 using data from both experiments.

For all two-way ANOVA analyses described above, effect sizes were estimated using partial eta-squared (η^2^_p_). When no significant interaction was detected, pairwise differences among factor levels were evaluated using Tukey’s *post hoc* test or the Games–Howell test when homogeneity of variances was not met. In cases of significant interactions, simple effects were explored through pairwise comparisons within each factor level, applying Bonferroni correction for multiple testing.

Analyses were conducted using IBM SPSS Statistics 26.0 (Illinois, USA) General Linear Model procedure, GLM) with a significance level of 0.05. Results are presented as means ± SD (standard deviation).

#### 2.8.3. Fatty Acids

Fatty acid composition data were treated as compositional data and therefore analyzed following a compositional data analysis framework. Prior to multivariate analyses, fatty acid proportions were transformed using the centered log-ratio (CLR) transformation to account for the constant-sum constraint and to avoid spurious correlations inherent to compositional datasets. Zero values in fatty acid compositional data were replaced using the multiplicative replacement method (δ = 0.65 × minimum non-zero value) prior to CLR transformation. The CLR transformation was applied to the full fatty acid matrix, including all identified fatty acids.

Principal component analysis (PCA) was then performed on the CLR-transformed data to explore patterns of variation in fatty acid profiles associated with exposure time, PHBV concentration, and temperature. PCA was conducted using the covariance matrix with *n* = 3 biological replicates per treatment group. Scores and loading patterns were examined to interpret sample clustering and the contribution of individual fatty acids to the main axes of variation. For clarity of interpretation, fatty acid loadings are presented as bar plots, highlighting variables with the highest absolute contributions to each principal component.

## 3. Results

### 3.1. Growth and Development

**Figure 1:**
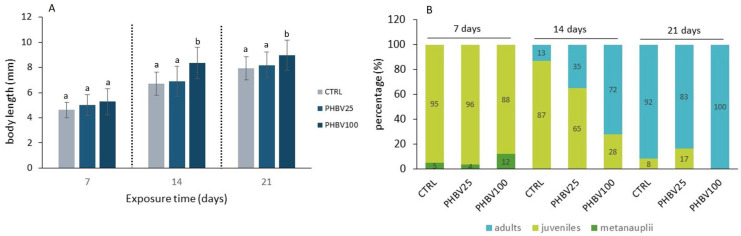
Body length (mm) (**A**) and developmental stage (%) (**B**) of *A. franciscana* exposed to different PHBV concentrations for 7, 14 and 21 days at 25 °C. Different letters indicate significant differences among concentrations within each time point (*p* < 0.05). Data after 14 days of exposure were adapted from Buzzi et al. [41].

Two-way ANOVA analysis showed that *Artemia* length in Exp. 1 was significantly affected by PHBV concentration and exposure time (F_2,210_ = 22.67, *p* < 0.001; F_2,210_ = 207.39, *p* < 0.001), and there was a significant interaction between both factors (F_4,210_ = 2.55, *p* < 0.05) (Table S1). Therefore, simple effects were analyzed using pairwise comparisons with multiple comparison correction. No significant differences in growth were observed at day 7. However, at days 14 and 21, growth was significantly higher in PHBV100 than in both the CTRL (day 14: *p* < 0.001; day 21: *p* = 0.001) and PHBV25 groups (day 14: *p* < 0.001; day 21: *p* = 0.022) (Figure 1A).

Regarding the developmental stage (Figure 1B), the results show that PHBV reduced development time. Specifically, a higher proportion of adults was observed with increasing PHBV concentrations at both 14 and 21 days after exposure. This effect was more pronounced after 21 days of exposure, with 100% of individuals in the PHBV100 treatment group having reached the adult stage, whereas in the CTRL group, 8% of the individuals were still juveniles.

**Figure 2:**
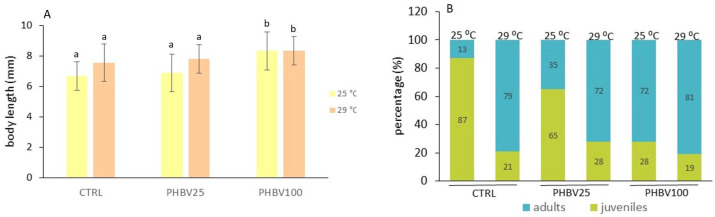
Body length (mm) (**A**) and development stage (%) (**B**) of *A. franciscana* exposed to different PHBV concentrations and temperatures for 14 days. Different letters indicate significant differences among concentrations (Tukey test, *p* < 0.05). Temperature had a significant overall effect (*p* < 0.001).

To further evaluate temperature effects (25 vs. 29 °C) on growth and development, data after 14 days of exposure were compared. Instar II-III (48 h old) organisms showed significant differences in body length at the beginning of the experiments (*p* < 0.001), with greater growth observed at 29 °C (0.89 ± 0.12 mm) compared to 25 °C (0.77 ± 0.07 mm). After 14 days of exposure, the two-way ANOVA test revealed no significant interaction between the main factors’ concentration and temperature exposure (F_2,140_ = 2.80, *p* = 0.065). Significant main effects were, however, observed for both factors: temperature (F_1,140_ = 10.96; *p* = 0.001) and concentration (F_2,140_ = 17.41, *p* < 0.001) (Table S2). Regarding concentration, better growth was observed at the highest PHBV concentration (PHBV100), as indicated by Tukey’s post hoc test (*p* < 0.05) (Figure 2A). In addition, temperature significantly influenced growth (*p* < 0.001), with higher values recorded at 29 °C compared to 25 °C.

Regarding developmental stage, a higher proportion of adults was observed at 29 °C than at 25 °C after 14 days. Increasing PHBV concentration was associated with a shift towards adult stages, an effect that was particularly evident at 25 °C (Figure 2B). At the highest concentration (PHBV100), adults predominated under both temperature treatments.

### 3.2. Changes in PHBV Particle Morphology and Size Following Ingestion

As previously described in Buzzi et al. [41], PHBV particles were characterized prior to exposure to confirm polymer identity and to assess the particle size distribution in suspension, ranging from 2 to 15 μm. This range falls within the reported ingestible size range for *A. franciscana*, with a considerable proportion of sub-2 μm particles also detected.

**Figure 3:**
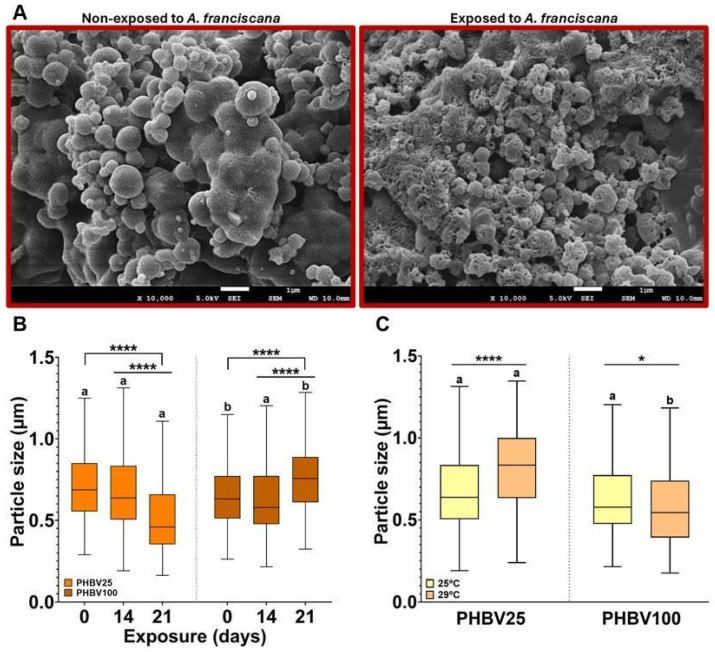
SEM micrographs illustrating the pristine PHBV prior to exposure (left) and particles retrieved from fecal pellets after ingestion (right) (**A**). Particle length distribution recovered from fecal pellets of *A. franciscana* exposed to different PHBV concentrations (**B**) for 0, 14 and 21 days at 25 °C and (**C**) under 25 °C and 29 °C at day 14. Different letters indicate significant differences among concentrations within each time point or temperature (*p* < 0.05). Asterisks indicate significant differences between time points or temperatures within each concentration (* (*p* < 0.05), **** (*p* < 0.0001)).

SEM images highlighted marked differences between pristine and ingested PHBV particles (Figure 3A). Non-exposed particles presented a uniform and compact morphology, while across all samples tested, ingested particles showed pronounced surface irregularities, including increased roughness and distorted shapes. Structural damage such as fractures or surface erosion was evident, with some particles partially associated with fecal material.

To complement the SEM analyses, Fourier Transform Infrared (FTIR) spectroscopy was performed on fecal pellets to evaluate potential changes in the polymer following gut passage. However, characteristic PHBV absorption bands could not be clearly resolved because the spectral contribution of the surrounding organic matter dominated the FTIR signal.

PHBV25 and PHBV100 particle length distributions (Figure S2) were centered at 0.6–0.8 µm and 0.5–0.7 µm, respectively, both with a right-skewed tail up to 1.7 µm and a low frequency of particles below 0.4 µm. In Exp. 1 at 14 days, both concentrations maintained distributions close to baseline; however, PHBV25 showed a narrower distribution, increased frequencies below 0.5 µm and a reduced upper tail. In contrast, PHBV100 exhibited a broader, more right-skewed distribution. By 21 days, PHBV25 distribution moved towards smaller lengths (peak at 0.4–0.5 µm), with an increase in small particles and a reduction in particles > 1 µm, whereas PHBV100 shifted towards larger lengths with a broader spread. In Exp. 2, PHBV25 at 29 °C showed a distribution shifted towards larger particle lengths (0.9–1.1 µm range) compared to 25 °C. Contrarily, PHBV100 at 29 °C showed a pronounced change from 25 °C, with a narrower distribution and a lower frequency of particles (0.4–0.6 µm range).

Mean particle lengths (±SE) for all experimental conditions are shown in Figure 3B (Exp. 1) and Figure 3C (Exp. 2). Normality and homoscedasticity tests indicated deviations from assumptions, which were not fully resolved after log-transformation. However, the analyses were considered robust (*n* > 200 measurements per condition), and two-way ANOVA tests were applied to assess the effects of the experimental factors on particle length.

(i) *Concentration* × *time*

As shown in Figure 3B, PHBV25 registered the highest and lowest mean particle length: 0.71 ± 0.02 µm at day 0 and 0.52 ± 0.01 µm at day 21, respectively. PHBV100 showed a decrease in mean values from 0.66 ± 0.02 µm at day 0 to 0.63 ± 0.02 µm at day 14, followed by an increase at 21 days (0.76 ± 0.02 µm).

Two-way ANOVA showed that particle size was significantly affected by exposure time (F_2,13_ = 4.84, *p* < 0.01) and concentration (F_1,13_ = 15.94, *p* < 0.0001), with a significant interaction between factors (F_2,13_ = 63.86, *p* < 0.0001). Partial eta-squared analysis indicated that variability was mainly explained by the interaction term (η^2^_p_ = 0.0921), followed by concentration (η^2^_p_ = 0.0125) and time (η^2^_p_ = 0.0076) (Table S1).

Following the significant interaction, simple effects were examined using Bonferroni-corrected pairwise comparisons (Figure 3B). Both concentrations showed significant differences (*p* < 0.0001) across time, particularly between 0 and 21 days and between 14 and 21 days. However, temporal patterns between concentrations were different: PHBV25 exhibited a progressive decrease in particle length over time; and PHBV100 showed an initial decrease from day 0 to 14, followed by an increase at day 21. When comparing concentrations within each time point, significant differences between PHBV25 and PHBV100 were observed at 0 (*p* < 0.05) and 21 days (*p* < 0.0001), while no significant differences were detected at 14 days.

(ii) *Concentration* × *temperature*

As shown in Figure 3C, PHBV25 at 29 °C exhibited the highest mean (0.802 ± 0.02 µm), while PHBV100 at 29 °C was the lowest (0.58 ± 0.02 µm). At 25 °C, the mean values were lower in PHBV25 (0.68 ± 0.015 µm) and PHBV100 (0.63 ± 0.02 µm) compared to PHBV25 at 29 °C.

Two-way ANOVA showed significant effects of both temperature (F_1,84_ = 4.55, *p* < 0.05) and concentration (F_1,84_ = 70.11, *p* < 0.0001). A significant interaction between temperature and concentration was also detected (F_1,84_ = 31.95, *p* < 0.0001). Partial eta-squared analysis indicated that concentration accounted for the largest proportion of the explained variability (η^2^_p_ = 0.077), followed by the interaction (η^2^_p_ = 0.037) and temperature (η^2^_p_ = 0.005) (Table S2).

Following the significant interaction, simple effects were examined using Bonferroni-corrected pairwise comparisons (Figure 3C). Within PHBV25, particle size was significantly higher at 29 °C compared to 25 °C (*p* < 0.0001). Within PHBV100, a smaller but significant difference between temperatures was also observed (*p* < 0.05). When comparing concentrations within each temperature, no significant differences were detected between PHBV25 and PHBV100 at 25 °C, whereas a highly significant difference was observed at 29 °C (*p* < 0.0001).

### 3.3. Biomarkers

The effects of different PHBV concentrations and exposure times on biomarkers in *Artemia* at 25 °C are described below.

#### 3.3.1. Catalase Activity (CAT)

**Figure 4:**
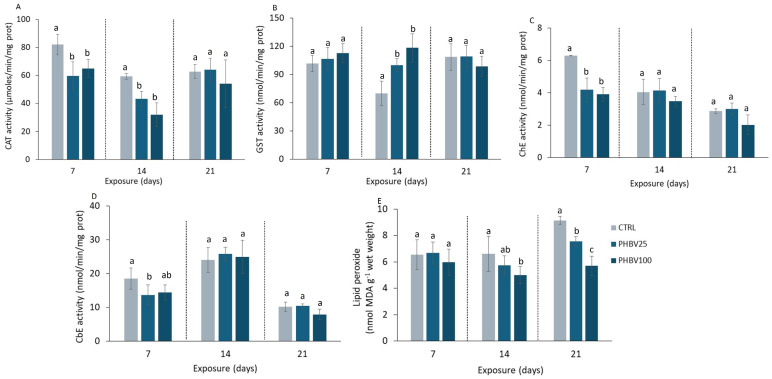
Biomarker responses of *A. franciscana* organisms exposed to different PHBV concentrations for 7, 14 and 21 days at 25 °C. (**A**) Catalase activity; (**B**) Glutathione-S-Transferase activity; (**C**) Cholinesterase activity; (**D**) Carboxylesterase activity; (**E**) Lipid peroxidation. Lower-case letters indicate significant differences between concentrations for each exposure time (*p* < 0.05). Lipid peroxidation data at 25 °C after 14 days of exposure were adapted from Buzzi et al. [41].

CAT activity was significantly affected by exposure time and PHBV concentration (Figure 4A). The highest value of CAT activity was observed in CTRL organisms after 7 days (82.13 ± 7.30 µmol min mg protein^−1^), whereas the lowest activity was recorded in organisms exposed to PHBV 100 mg·L^−1^ after 14 days (31.90 ± 8.42 µmol min mg protein^−1^) (Figure 4A). The two-way ANOVA test revealed significant main effects of exposure time (F_2,22_ = 51.28; *p* < 0.001) and PHBV concentration (F_2,22_ = 28.62, *p* < 0.001), as well as a significant interaction between both factors (F_4,22_ = 5.18, *p* = 0.004). Partial eta-squared analysis revealed that variability was similarly explained by both factors, exposure time (η^2^_p_ = 0.823) and PHBV concentration (η^2^_p_ = 0.722) (Table S1). Following the significant interaction, simple effects were examined using pairwise comparisons with multiple comparison correction. This analysis revealed a decrease in CAT activity in organisms exposed to PHBV at days 7 and 14 (*p* < 0.05), while no differences were observed after 21 days of exposure (*p* > 0.05) (Figure 4A).

#### 3.3.2. Glutathione-S-Transferase Activity (GST)

Organisms exposed to 100 mg·L^−1^ PHBV exhibited the highest GST activity after 14 days (118.34 ± 15.23 nmol min^−1^ mg protein^−1^), whereas the CTRL group showed the lowest values (69.85 ± 13.04 nmol min^−1^ mg protein^−1^) (Figure 4B). GST activity was not significantly affected by exposure time (F_2,21_ = 3.33, *p* = 0.056). In contrast, significant differences were observed among PHBV concentrations (F_2,21_ = 7.80, *p* = 0.003), and a significant interaction between concentration and exposure time was detected (F_4,21_ = 8.47, *p* < 0.001). Partial eta-squared analysis indicated that variability was mainly explained by PHBV concentration (η^2^_p_ = 0.426), followed by exposure time (η^2^_p_ = 0.241) (Table S1). Post hoc pairwise comparisons revealed significant differences in GST activity only after 14 days of exposure, with higher activity in the PHBV25 and PHBV100 treatments than in the CTRL (*p* < 0.001) (Figure 4B).

#### 3.3.3. Cholinesterase Activity (ChE)

The highest ChE activity was observed in the CTRL group after 7 days (6.30 ± 0.01 nmol min mg protein^−1^), while the PHBV100 group exhibited the lowest activity after 21 days of exposure (2.02 ± 0.6 nmol min mg protein^−1^) (Figure 4C). Two-way ANOVA revealed that ChE activities varied significantly with both PHBV concentration and exposure time (F_2,15_ = 17.69, *p* < 0.001; F_2,15_ = 45.53, *p* < 0.001), with a significant interaction between both factors (F_4,15_ = 4.74, *p* = 0.011). Partial eta-squared analysis indicated that variability was similarly explained by exposure time (η^2^_p_ = 0.859) and concentration (η^2^_p_ = 0.702) (Table S1). Simple effects analysis, based on pairwise comparisons with multiple comparison correction, revealed a significant decrease in ChE activity in PHBV25 (*p* < 0.001) and PHBV100 (*p* < 0.001) compared to the CTRL after 7 days of exposure (Figure 4C).

#### 3.3.4. Carboxylesterase Activity (CbE)

The highest CbE activity was found in the PHBV25 group at day 14 (25.75 ± 2.05 nmol min mg protein^−1^), while organisms from PHBV100 showed the lowest values after 21 days of exposure (7.88 ± 1.50 nmol min mg protein^−1^) (Figure 4D). The two-way ANOVA test showed that CbE activity varied significantly with concentration (F_2,21_ = 3.49, *p* = 0.049) and exposure time (F_2,21_ = 144.25, *p* < 0.001), and a significant interaction between both factors was also detected (F_4,21_ = 3.46, *p* = 0.026). Partial eta-squared analysis revealed that most of the variability was explained by exposure time (η^2^_p_ = 0.932), followed by concentration (η^2^_p_ = 0.250) (Table S1). Simple effects using pairwise comparisons with multiple comparison correction showed that after 7 days of exposure, CbE activity in *Artemia* was significantly lower in the PHBV25 group than in the CTRL (*p* < 0.036), while no significant differences were observed between PHBV25 and PHBV100 (Figure 4D).

#### 3.3.5. Lipid Peroxidation (LPO)

The highest LPO was detected in the CTRL group after 14 days (9.14 ± 0.30 nmol MDA g^−1^ wet weight), whereas organisms from PHBV100 showed the lowest values (4.99 ± 0.65 nmol MDA g^−1^ wet weight) (Figure 4E). The effects of both factors, concentration and exposure time, were statistically significant (F_2,20_ = 14.68, *p* < 0.001; F_2,20_ = 9.32, *p* < 0.001), as well as their interaction (F_4,20_ = 3.17, *p* = 0.036). Partial eta-squared analysis revealed that variability was similarly explained by both factors, concentration (η^2^_p_ = 0.595) and exposure time (η^2^_p_ = 0.482) (Table S1). Post hoc pairwise comparisons revealed significant differences among PHBV concentrations after 14 and 21 days of exposure. After 14 days, MDA levels in the PHBV100 group were significantly lower than in the CTRL (*p* < 0.018). At day 21, MDA levels decreased progressively from the CTRL to PHBV100, with significant differences observed between the CTRL and PHBV25 (*p* = 0.014), the CTRL and PHBV100 (*p* < 0.001), and PHBV25 and PHBV100 (*p* = 0.013) (Figure 4E).

To further evaluate temperature effects (25 vs. 29 °C) on biomarker responses, data after 14 days of PHBV exposure were compared.

#### 3.3.6. Catalase Activity (CAT)

**Figure 5:**
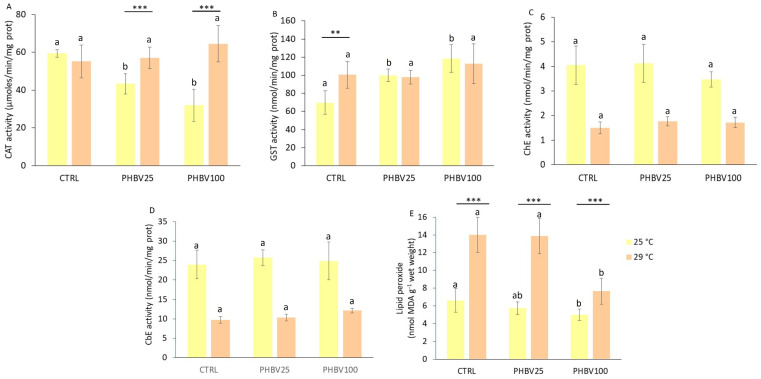
Biomarker responses of *A. franciscana* organisms exposed to different PHBV concentrations and temperatures for 14 days. (**A**) Catalase activity; (**B**) Glutathione-S-Transferase activity; (**C**) Cholinesterase activity; (**D**) Carboxylesterase activity; (**E**) Lipid peroxidation. Different letters indicate significant differences among concentrations within each temperature (*p* < 0.05). Asterisks indicate significant differences between temperatures within each concentration ** (*p* < 0.01), *** (*p* < 0.001)). For ChE and CbE, temperature had a significant overall effect (*p* < 0.001).

Considering the effects of temperature and concentration on CAT activity after 14 days, the two-way ANOVA test showed a significant main effect of both factors, temperature (F_1,24_ = 28.93, *p* < 0.001) and concentration (F_2,24_ = 4.49, *p* < 0.022). In addition, the interaction between both factors was significant (F_2,24_ = 16.61, *p* < 0.001). Consequently, partial eta-squared analysis revealed that variability was mainly explained by temperature (η^2^_p_ = 0.547), followed by the concentration factor (η^2^_p_ = 0.272) (Table S2). Following the significant interaction, simple effects were examined using pairwise comparisons with multiple comparison correction. This analysis showed lower CAT activities in *Artemia* exposed to 25 (*p* = 0.005) and 100 mg·L^−1^ PHBV (*p* < 0.001) at 25 °C compared to those exposed at 29 °C (Figure 5A). Significant differences among concentrations at 25 °C were also observed, with the PHBV25 and PHBV100 groups showing lower activity than the CTRL (CTRL and PHBV25 *p* < 0.005; CTRL and PHBV100 *p* < 0.001; PHBV25 and PHBV100 *p* = 0.055).

#### 3.3.7. Glutathione-S-Transferase Activity (GST)

Two-way ANOVA revealed that GST activity in *Artemia* exposed to PHBV for 14 days was not significantly affected by temperature (F_1,24_ = 2.24, *p* = 0.148). In contrast, significant differences were observed among PHBV concentrations (F_2,24_ = 11.42, *p* < 0.001), and a significant interaction between both factors was also detected (F_2,24_ = 4.94, *p* = 0.016). Partial eta-squared analysis indicated that variability was mainly explained by concentration (η^2^_p_ = 0.488), followed by temperature (η^2^_p_ = 0.085) (Table S2). Following the significant interaction, simple effects were examined using pairwise comparisons with multiple comparison correction. This analysis showed that GST activity was significantly higher in CTRL organisms exposed to 29 °C than those exposed to 25 °C (*p* < 0.002), while no significant effect of temperature was detected in the PHBV25 and PHBV100 groups (Figure 5B). Significant differences among PHBV concentrations at 25 °C were observed; specifically, GST activity was higher in the PHBV25 and PHBV100 groups than in CTRL organisms (CTRL and PHBV25 *p* = 0.008; CTRL and PHBV100 *p* < 0.001; PHBV25 and PHBV100 *p* = 0.153), whereas no significant differences were observed at 29 °C.

#### 3.3.8. Cholinesterase Activity (ChE)

After 14 days of exposure to different temperatures and PHBV concentrations, no differences in ChE activity were observed among *Artemia* exposed to the different concentrations (F_2,21_ = 1.27, *p* > 0.302). In contrast, ChE activity differed significantly according to exposure temperature (F_1,21_ = 218.72, *p* < 0.001), with higher activity at 25 °C compared to 29 °C (*p* < 0.001) (Figure 5C). No significant interaction was detected between concentration and temperature (F_2,21_ = 1.87, *p* > 0.179) (Table S2).

#### 3.3.9. Carboxylesterase Activity (CbE)

CbE activity showed significant differences among *Artemia* organisms exposed to different temperatures (F_1,23_ = 365.46, *p* < 0.001), with higher activity at 25 °C than at 29 °C (*p* < 0.001). In contrast, no significant variation in CbE activity was observed in relation to PHBV concentration (F_2,23_ = 2.80, *p* = 0.081), nor was there a significant interaction between temperature and concentration (F_2,23_ = 2.38, *p* = 0.115) (Table S2).

#### 3.3.10. Lipid Peroxidation (LPO)

After 14 days of exposure, LPO levels were significantly affected by both temperature (F_1,23_ = 141.43, *p* < 0.001) and PHBV concentration (F_2,23_ = 22.78, *p* < 0.001). In addition, a significant interaction between temperature and concentration was observed (F_2,23_ = 5.80, *p* = 0.009). In this case, the partial eta-squared analysis showed that variability in LPO was mainly explained by temperature (η^2^_p_ = 0.860), followed by PHBV concentration (η^2^_p_ = 0.665) (Table S2). Simple effect comparisons with multiple comparison correction analysis revealed significantly higher MDA concentrations at 29 °C than at 25 °C in both PHBV concentrations (*p* < 0.001). Then, at 25 °C, LPO was significantly lower in organisms exposed to PHBV100 compared to the CTRL (*p* < 0.044). Similarly, at 29 °C, MDA levels were significantly lower in the PHBV100 group than in both the CTRL and PHBV25 groups (*p* < 0.001) (Figure 5E).

### 3.4. Fatty Acid Profiles

**Figure 6:**
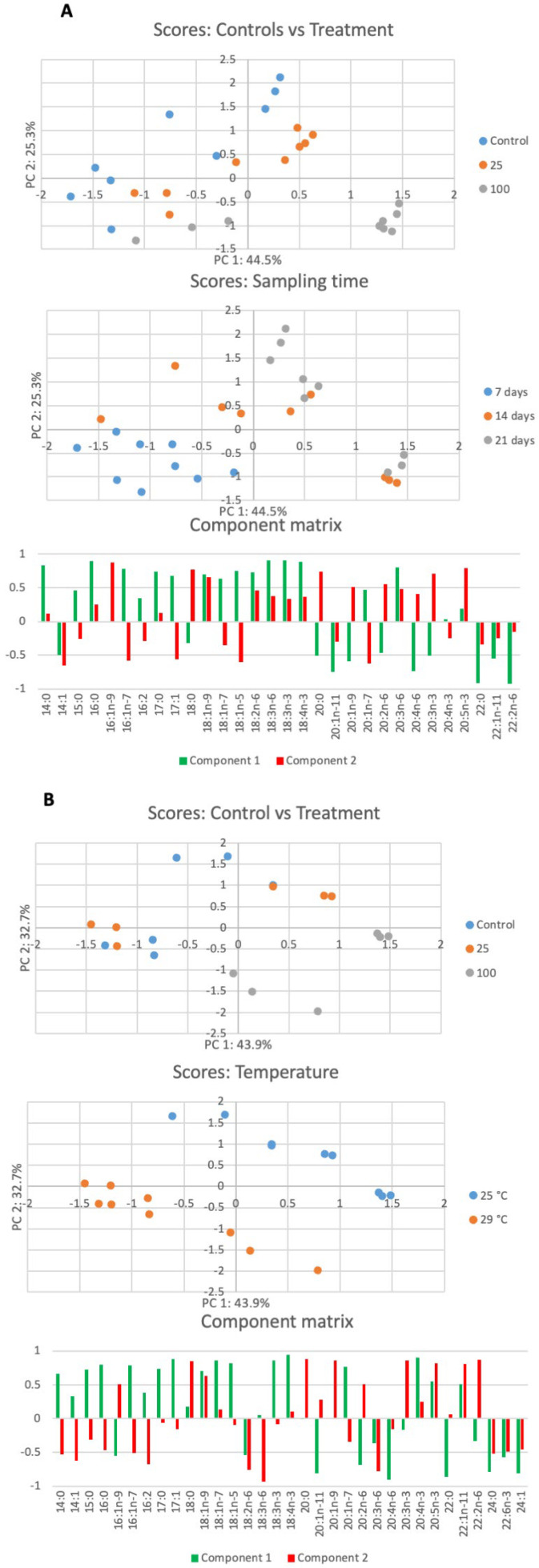
Principal component analysis (PCA) of centered log-ratio (CLR)-transformed fatty acid profiles of *A. franciscana* exposed to PHBV microplastics. (**A**) PCA score plots showing the separation of samples according to PHBV concentration (control, 25 and 100) across the different sampling times. (**B**) PCA score plots illustrating the separation of the same PHBV treatments as a function of temperature (25 °C and 29 °C). Percentages on the axes indicate the variance explained by PC1 and PC2. Bar plots below each score plot represent the loadings derived from the component (loading) matrix for PC1 and PC2, highlighting the fatty acids that contribute most strongly to sample discrimination along each axis. The loading histograms summarize the direction and relative contribution of individual fatty acids to the principal components, providing the basis for interpreting temporal, concentration-dependent, and temperature-driven patterns observed in the PCA scores; 25 and 100: PHBV25 and PHBV100.

Inspection of the PCA score plots, together with the corresponding fatty acid loading bar plots (Figure 6), shows that the main sources of variability in fatty acid composition are captured by distinct principal components. The first principal component (PC1), which explains the largest proportion of variance, primarily separates samples according to time of exposure and, to a lesser extent, temperature. The associated PC1 loading bar plot indicates that this separation is driven by coordinated variation across a wide range of fatty acids, including saturated fatty acids (14:0, 16:0 and 18:0), monounsaturated fatty acids (16:1 and 18:1n-9), and several polyunsaturated fatty acids (PUFAs).

In contrast, variation along the second principal component (PC2) captures additional differences in fatty acid composition that are not as clearly associated with a single experimental factor. Although some distinction between treatment groups is observable, separation of organisms exposed to the highest PHBV concentration (PHBV100) from the control (CTRL) and PHBV25 groups is not consistent across all conditions. Loadings on PC2 are mainly associated with several long-chain PUFAs, particularly n-6 and n-3 fatty acids such as 18:2n-6, 20:4n-6, 20:5n-3 and 22:6n-3, as shown in the PC2 loading bar plot; however, contributions from other fatty acids are also evident.

Overall, the PCA indicates that variability related to exposure time and, to a lesser extent, temperature, accounts for most of the variation in fatty acid profiles, whereas treatment effects are present but less clearly resolved and involve more subtle compositional changes, including shifts in specific PUFAs.

### 3.5. Histology

**Figure 7:**
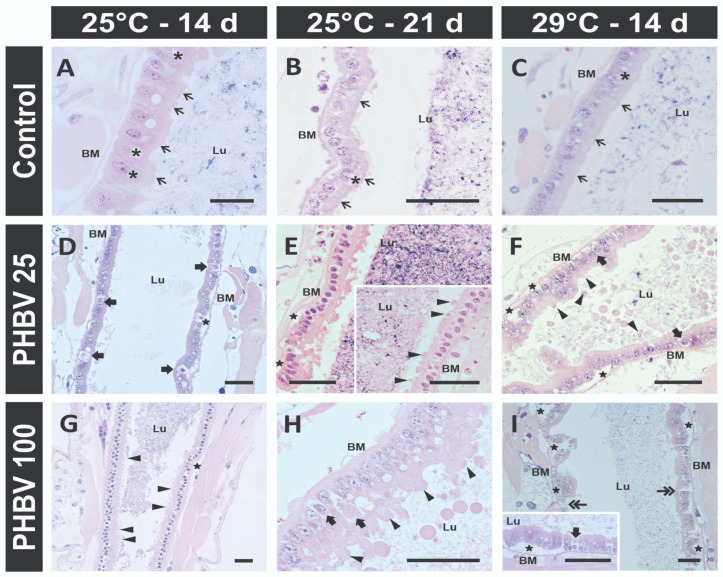
Micrographs of the digestive tract of *A. franciscana* from Exp. 1, after 14 days (**D**,**G**) and 21 days (**E**,**H**) of exposure to PHBV at 25 °C, and from Exp. 2, after 14 days of exposure to PHBV at 29 °C (**F**,**I**). (**A**–**C**) Control organisms (not exposed to PHBV); (**D**–**F**) organisms exposed to 25 mg⋅L^−1^ PHBV (PHBV25); (**G**–**I**) organisms exposed to 100 mg⋅L^−1^ PHBV (PHBV100). Figure references: arrowhead, blebbing cells; asterisk, supranuclear vesicles in enterocytes; BM, epithelial basal membrane; double-headed arrow, disruption of the digestive epithelium; Lu, digestive tract lumen; star, detachment of epithelial cells; thin arrow, microvilli of enterocytes; thick arrow, edematous cells. Scale bar: 50 μm. Figures (**A**,**D**,**G**) are adapted from Buzzi et al. [41].

The histopathological evaluation of the digestive tract of *A. franciscana* was performed in samples from Exp. 1 (after 14 and 21 days at 25 °C) and from Exp. 2 (after 14 days at 29 °C), exposed to two different PHBV concentrations (25 and 100 mg·L^−1^, PHBV25 and PHBV100, respectively) (Figure 7).

In all treatments, control sections of *A. franciscana* (Figure 7A–C) showed that the digestive tract epithelium was simple columnar, with enterocytes exhibiting basal euchromatic nuclei, prominent nucleoli and microvilli, and acidophilic granular cytoplasm. Supranuclear vesicles of varying sizes were also observed.

In both PHBV treatments (PHBV25 and PHBV100), at 25 °C for 14 and 21 days and at 29 °C for 14 days, alterations in the cytoarchitecture of the digestive epithelium were observed (Figure 7D–I) compared with the CTRL. The main alterations included the presence of cells exhibiting blebbing (Figure 7E–H) and the detachment of epithelial cells (Figure 7D–G,I). Some of the epithelial cells showed edema, with swelling and a pale, enlarged cytoplasm (Figure 7D,F,H,I), along with hypertrophied epithelial cells (Figure 7E–I) and epithelial hyperplasia (Figure 7F–H). In some cases, degeneration and disruption of the digestive epithelium were observed (Figure 7I).

**Figure 8:**
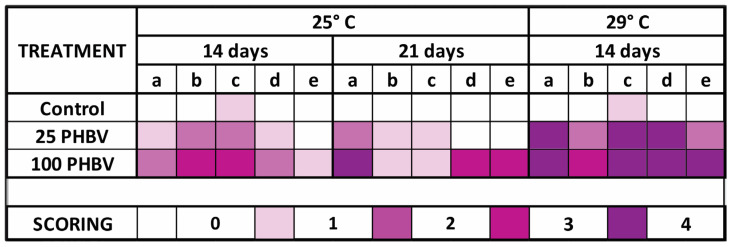
Semi-quantitative evaluation of the damage level in the digestive tract of *A. franciscana* from Exp. 1, after 14 days and 21 days of exposure to PHBV at 25 °C, and from Exp. 2, after 14 days of exposure to PHBV at 29 °C. Scale: 0: normal; 1: mild (less than 25% of damage); 2: moderate (less than 50% of damage); 3: severe (less than 75% of damage), and 4: very severe (more than 75% of damage). Damage indicators: presence of blebbing cells (a), epithelium cell disruption (b), detachment of the epithelium (c), presence of edematous enterocytes (d) and cell hyperplasia (e).

The semi-quantitative evaluation of the damage level according to treatment concentration and temperature is shown in Figure 8.

## 4. Discussion

In this study, we investigated the effects of PHBV MPs on the physiological, biochemical, and histological responses of the brine shrimp *A. franciscana* under different exposure conditions, including temperature, duration, and concentration. *Artemia* species are adapted to a wide range of environmental conditions, including broad fluctuations in temperature (6–40 °C), salinity, and ionic composition of their habitat. Although both temperature and salinity significantly affect survival and growth, the effect of temperature is more pronounced [51].

The body length and developmental stage of *A. franciscana* were examined in order to understand the impact of PHBV microparticles on its growth.

Our study demonstrated an increase in *Artemia* length and faster development following exposure to PHBV, specifically at the highest bioplastic concentration (PHBV100). This was similar to the effects observed by Afifah et al. [6] in the same species exposed to lower concentrations of biodegradable Poly-3-hydroxybutyrate (P3HB) after 14 days. On the contrary, Biandolino et al. [52] found that sublethal concentrations of PLA (5 and 10 mg·L^−1^) resulted in lower growth and reproduction success in the marine amphipod *Gammarus aequicauda* after 60 days. Also, Procházková et al. [53] showed that P3HB (12.5 and 25 mg·L^−1^ P3HB) caused a slight inhibition in the growth of *Daphnia magna* after 21 days of exposure. According to these authors, besides MP concentration, particle size also plays an important role since a more pronounced effect was observed in *D. magna* with smaller particles, probably due to higher bioaccumulation. The size of PHBV microparticles used in this study ranged from 2 to 15 μm [41], and despite being smaller than the particles used by Procházková et al. [53], the feeding behavior and ingestion in *Artemia* did not change [41]. Moreover, the marked differences between pristine and ingested PHBV particle morphology suggest that gut passage may involve mechanical abrasion and potentially other biological processes [41], although the relative contribution of these mechanisms cannot be determined from the present data. The observed changes in particle morphology and size distribution could therefore result from physical fragmentation during gut transit, biological processing, or a combination of both. This may also be linked to the non-selective feeding behavior of *Artemia*, which preferentially grazes on particles smaller than ~50 μm, with optimal range size between 6.8 and 27.5 [54], and may adjust ingestion, absorption efficiency, and gut passage time to optimize energy intake [55,56], potentially influencing the size distribution of particles observed in fecal pellets.

*Artemia*, through the continuous beating of its appendages (thoracopods), generates feeding currents in order to capture any type of suspended particle, whether organic or inorganic [57], including MPs and nanoplastics [40,48]. These characteristics explain the consistent ingestion of PHBV observed in our previous work [41] and in the present study. Moreover, continuous particle uptake during feeding, potentially contributes to the physiological and biochemical responses reported here. Further studies combining particle characterization with analyses of digestive enzyme activity and polymer degradation products are needed to clarify the mechanisms underlying these changes.

The present results highlight that fragmentation dynamics of PHBV particles depend not only on ingestion but also on exposure time and bioplastic concentration. Under lower-concentration conditions, a progressive tendency towards the generation of smaller particle lengths over time was observed, suggesting more efficient processing of the ingested material by the organisms. Conversely, at higher concentrations, the pattern differed: an initial decrease in particle length was followed by an increase after prolonged exposures. This may indicate saturation of the digestive capacity and/or compromised efficiency in particle fragmentation. This differential behavior is consistent with the idea that the capacity of filter-feeding organisms to process MPs is limited and can be impaired under high particle loads, as previously described in planktonic crustaceans [58].

The modulation of MP fragmentation by exposure concentrations and exposure time observed in Antarctic krill (*Euphausia superba*) may have important implications for other planktonic crustaceans such as *A. franciscana*. In krill, low LDPE MP concentrations promote efficient fragmentation, resulting in a higher proportion of smaller particles in fecal pellets, whereas high concentrations progressively impair this capacity, leading to increased egestion of intact particles over time [58]. A similar mechanism may operate in *A. franciscana*, where long-term high-concentration PHBV (PHBV100) could saturate digestive processing and reduce particle breakdown efficiency. Moreover, short-term exposure to 25 and 100 mg·L^−1^ PHBV resulted in similar particle sizes in fecal pellets, while long-term 25 mg·L^−1^ PHBV exposure led to the production of smaller-sized microparticles. This is particularly relevant in the context of our experimental conditions, as it suggests that the size distribution of egested MPs is not only a function of the ingested material but also of organismal processing capacity under different exposure regimes. Consequently, *Artemia* may act as a generator of different sizes of secondary MPs, depending on environmental concentrations and exposure time, potentially contributing to the redistribution of particle sizes in the environment. This process has relevant ecological implications, as the availability of smaller particles might increase their bioavailability for other organisms, thereby facilitating their transfer to higher trophic levels.

The enhanced development observed in *Artemia* exposed to PHBV is consistent with the findings reported for the Chinese mitten crab, *Eriocheir sinensis*, whose larvae fed *Artemia* enriched with Poly-b-hydroxybutyrate (PHB) exhibit higher survival and better development [59]. The same results were reported by Kim et al. [60] in the Pacific white shrimp (*Penaeus vannamei*) post-larvae fed *Artemia* enriched with PHB. Jaapar et al. [24] found no differences in development time from egg to adult in the copepod *Nitokra lacustris pacifica* exposed to PHBV (700 microbeads·mL^−1^), but it was shorter than in organisms exposed to PS. As suggested by Buzzi et al. [41], PHBV may act in synergy with microalgae or provide a nutritional contribution and could potentially exert therapeutic-like effects, as reported for PHB [61], which may contribute to the enhanced growth and development observed in exposed organisms, although the mechanisms responsible for these effects remain to be elucidated.

At higher temperatures (29 °C), brine shrimp exhibited greater growth and a higher proportion of adults compared to those at 25 °C. Previous studies have reported similar findings: *Artemia* grew faster and attained sexual maturity sooner at elevated temperatures (20 °C, 25 °C and 30 °C) [62]. This study also documented increased mortalities positively correlated with increasing water temperatures; this was likewise observed in the present study. In this sense, the long-term experiment at 29 °C (Exp. 2) could have selected only the most resistant organisms. Conversely, Afifah et al. [6] found a significant decrease in the growth of *A. franciscana* at 30 °C compared to 26 °C after 14 days, probably because reduced oxygen in water induces stress in the organisms, affecting food intake and, ultimately, growth.

Additionally, body length and development stage were further enhanced in *Artemia* exposed to 100 mg·L^−1^ PHBV at both temperatures; however, the increase in growth was not associated with the combined effect of temperature and bioplastic concentration, as their interaction was not significant. Afifah et al. [6] found that *A. franciscana* growth was significantly affected by temperature and MP concentration but not by the type of bioplastic (PHA, PLA and PBS). Regarding conventional polymers, Han et al. [35] observed that PS and temperature act as combined stressors affecting *Artemia* growth and survival after 14 days of exposure. Considering that increased temperature leads to higher oxygen consumption in *Artemia* [63], which reflects elevated metabolic demand, Han et al. [35] suggested that this may enhance feeding activity and MP ingestion, thereby intensifying PS toxicity. In line with this, our results indicate that temperature may also modulate the processing of ingested PHBV particles. Differences in particle length between 25 and 29 °C suggest that warmer conditions influence digestive processing and therefore fragmentation efficiency, probably due to temperature-driven changes in metabolic activity, feeding rates, or gut transit time.

The histology of the digestive tract in control individuals of *A. franciscana* at both temperatures (25 and 29 °C) is consistent with previous descriptions for the genus *Artemia* [41,64]. However, notable microarchitecture alterations were observed in the digestive tract of *A. franciscana* exposed to PHBV from all the experiments. As reported by Buzzi et al. [41], and similar to the results of Wang et al. [65], although *Artemia* seems to rapidly eliminate PHBV microparticles after ingestion, these could still exert adverse impacts on intestinal tissues.

Overall, temperature seems to act as the main factor influencing the response. At a given PHBV concentration, more pronounced adverse effects were consistently observed at 29 °C compared with 25 °C. In contrast, extending the exposure time from 14 to 21 days at 25 °C did not result in a comparable increase in damage indicators. Although in this study *Artemia* was exposed to high concentrations of bioplastic that may exceed environmentally relevant levels, the histological results are similar to those reported by Han et al. [35] with conventional MPs. These authors showed that exposure of *Artemia* at 30 °C to 2 mg·L^−1^ of PE MPs for 14 days reduced the number of intestinal microvilli and epithelial cells. They also proposed that elevated temperatures may increase MP ingestion, metabolic activity, and energy expenditure, thereby exacerbating the toxic effects of MPs. However, the effect of exposure time was not considered in their study. According to Suman et al. [66], epithelial deformation and cell derangement observed in the *Artemia* gut after 48 h or 14 days of exposure to PS MPs (100 and 1 mg·L^−1^, respectively) may be related to the generation of reactive oxygen species (ROS). Moreover, digestive tract alterations may cause digestive dysfunction, reducing energy metabolism and nutrient absorption [66,67]. Otherwise, Lei et al. [68] observed that the extent of intestinal lesions is primarily driven by the size and shape of the microparticles rather than the chemical composition of the polymer.

In our study, when temperature and exposure time were held constant, higher PHBV concentrations seemed to be associated with an increase in histopathological indicators, although this effect was apparently less marked than that of temperature. The smaller particle size observed in fecal pellets at 29 °C may increase the potential for interaction with biological tissues, potentially contributing to the greater histological damage observed, particularly in the PHBV100 group. Finally, changes in gut microbiota in relation to histopathological lesions are not discarded [69].

In the present study, the effects of PHBV after long-term exposure were observed on key enzymes involved in several physiological processes, including the cellular antioxidant system (CAT and GST), neurotoxicity markers (ChE), xenobiotic biotransformation (CbE), and lipid peroxidation (LPO). The responses of these biochemical markers in *Artemia*, whether increases or decreases in activity, were driven by the combined effects of bioplastic concentration and exposure time. In the case of CAT, ChE and CbE, the variability was mostly explained by exposure time, while in the case of GST and LPO, it was explained by PHBV concentration.

Several studies have shown that MP exposure causes oxidative stress, which quickly activates antioxidant defenses. Previous works demonstrate an increase in CAT activity in *Artemia* spp. after short-term MP exposure [40,67,70]. This enzyme facilitates the removal of hydrogen peroxide (H_2_O_2_), which is metabolized to molecular oxygen (O_2_) and water [71]. Usually, an increase in antioxidant defenses occurs due to elevated ROS levels. In contrast, in the present study, CAT activity decreased in *Artemia* after 7 and 14 days of PHBV exposure, suggesting that this enzyme was not a primary antioxidant defense mechanism involved in the response to PHBV MPs, or that its activity was suppressed as a result of an excessive influx of superoxide radicals [72,73]. As reported by Yu et al. [20] in *E. sinensis* exposed to PS for 21 days, the present results show that CAT inhibition may be associated with the energy cost of oxidative stress response induced by MP exposure. Another study showed that MPs derived from PE bags and paper cups form a complex with CAT and cause a reduction in ∝-helical contents and an increase in random coils, inhibiting its activity [74]. Although this work was conducted on bovine liver CAT, a similar effect of PHBV on *Artemia* CAT cannot be ruled out.

We observed a significant increase in GST activity in *Artemia* after 14 days of bioplastic exposure. GST is a phase II detoxification enzyme involved in pollutant biotransformation in aquatic organisms and is also responsive to oxidative stress [71]. This response likely reflects a protective mechanism against ROS accumulation when other antioxidant defenses, such as CAT, fail, with GST assisting in the conversion of peroxides into less toxic hydroxyl compounds [40,73,75]. Similar results were found by Jeyavani et al. [67] in *A. salina* juveniles exposed to polypropylene MPs for 48 h and by Peixoto et al. [40] in *A. franciscana* juveniles exposed to low concentrations of fluorescent red polymer microspheres. Papadopoulos et al. [76] found that GST expression and, consequently, its activity decrease as *A. salina* grows, being higher 48 h after hatching and lower at the adult stage. These authors suggest that this increase would compensate for the decrease in GST affinity towards GSH. This partially explains the differences in GST activity found between the CTRLs.

The antioxidant defense system comprises multiple interconnected pathways and enzymes; therefore, observed responses should be interpreted with caution. Recent studies have shown that exposure to bioplastics, such as PLA and PHB, can modulate multiple components of the antioxidant defense system in aquatic invertebrates such as *D. magna* [77] and *Mytilus edulis* [78]. Although other antioxidant enzymes, such as superoxide dismutase (SOD) and glutathione peroxidase (GPx), were not assessed in the present study, their involvement in the response of *A. franciscana* to PHBV exposure cannot be excluded. Therefore, the CAT and GST responses observed here likely represent only part of a broader antioxidant response network. In addition, lipid peroxidation was assessed in the present study as a complementary indicator of oxidative stress, providing information on whether PHBV exposure induced oxidative damage to membrane lipids. The opposite responses observed for CAT and GST activities suggest a complex regulation of oxidative stress defense.

As reported in our previous study [41], exposure of *A. franciscana* to PHBV was associated with a decrease in LPO after 14 days. In the present study, these changes in LPO were not only maintained but were more significant after 21 days of exposure to the bioplastic. Similar to the findings of De Felice et al. [78] in *D. magna* exposed to PLA, the absence of oxidative damage after long-term exposure, as indicated by LPO levels, suggests that the organisms activated effective antioxidant defenses that prevented the harmful effects of prooxidant species generated by PHBV MP exposure. We speculated that *Artemia* might develop an adaptive response to MP stress over time, allowing CAT and GST activities to return to normal levels after long-term exposure. The decrease in LPO levels in organisms exposed to PHBV after 14 and 21 days indicated that the cells were not subjected to oxidative damage. The increase in GST activity likely helped protect the cells from oxidative stress and prevented MDA formation, thereby indirectly preserving cell membrane integrity [79,80]. Moreover, long-term exposure to MPs is sufficient to trigger adaptive responses in organisms, leading to an increase in enzymes capable of neutralizing reactive species responsible for biomolecule oxidation and lipid peroxidation [81].

Correia et al. [82] reported that total PUFA levels increased progressively with age in the amphipod *Gammarus lacusta*, contributing to higher LPO in older organisms. In contrast, despite age-related changes in fatty acid composition in *Artemia*, PUFA levels and LPO remained unchanged.

Previous studies have demonstrated that PHB and its degradation product, β-hydroxybutyrate (β-HB), can be metabolized by crustaceans as an energy source and are associated with improved survival and reduced oxidative stress [59,60,83]. In this context, Buzzi et al. [41] suggested that PHBV degradation products could potentially contribute to energy metabolism and help reduce oxidative pressure, enabling *A. franciscana* to maintain or even increase saturated fatty acid levels, resulting in membranes that are more stable and less prone to peroxidation. In line with this previous work, the present findings support the idea that PHBV does not induce a classical stress response characterized by increased oxidative damage. Further studies are required to determine whether PHBV degradation products contribute to energy metabolism or oxidative stress modulation in *A. franciscana*.

In relation to the ChE and CbE enzymes, both activities decreased after short-term exposure (7 days) and then returned to normal levels. Decreased activities indicate a neurotoxic effect and reduced capacity in MP detoxification, respectively. However, no deleterious impacts on development, feeding behavior and mortality were observed in *Artemia* exposed to PHBV [41], probably related to the tolerance of *A. franciscana* to ChE inhibition by micro- and nanoplastics [40,48]. Also, this apparent dissociation could reflect several non-exclusive mechanisms, including the existence of compensatory physiological processes or insufficient inhibition to trigger organism-level effects. Also, this apparent dissociation could indicate the absence of a clear organism-level effect, reflecting non-mutually exclusive mechanisms, including compensatory physiological processes or insufficient inhibition.

Miksch et al. [84] revealed an increase in the enzymatic activity of short-chain carboxylesterases together with no oxidative stress response in rockpool shrimp (*Palaemon elegans*) fed algal flakes coated with PHBV. This was not observed in the present study, possibly due to the rapid egestion of PHBV microparticles, which may limit their residence time in the digestive tract and consequently reduce the extent of polymer degradation [41]. In addition to the role of CbE in detoxification functions, this group of enzymes hydrolyzes methyl farnesoate, a molt-regulating hormone in crustaceans [85,86]. Consistent with the findings of Peixoto et al. [40] in *A. franciscana* exposed to fluorescent red polymer microspheres, no abnormal development was observed after either short- or long-term exposure to PHBV in the present study. Therefore, the observed enzymatic changes may represent sublethal physiological adjustments that were not translated into measurable effects on growth or development during the exposure period.

In addition, CbE is involved in xenobiotic detoxification processes and may contribute to buffering neurotoxic effects, as described for organophosphate compounds in invertebrates [71,87]. Further studies incorporating other behavioral endpoints would be necessary to clarify the functional significance of both enzymatic responses. Finally, the gradual recovery of ChE and CbE activities to CTRL levels with increasing exposure time (14 and 21 days) may be explained by adaptative responses of the organism [80]. However, the underlying mechanism of recovery in *A. franciscana* requires further investigation.

Warming affects the behavior and bioavailability of MPs, thereby modifying their toxicity to aquatic organisms. To assess temperature-dependent effects on biomarkers, data obtained after 14 days of exposure at 25 °C and 29 °C were combined and analyzed. The activities of ChE and CbE were not influenced by the combination of temperature and PHBV concentration; only the increase in temperature affected their activity, which was lower at 29 °C. Despite unclear molecular mechanisms, MPs are thought to disrupt nervous system function in zooplankton, impairing key behaviors such as feeding and vertical migration [88]. Although neither feeding behavior nor mortality was analyzed in the exposure of *Artemia* to PHBV at 29 °C, a higher mortality was generally observed, probably associated with the lower activity of ChE. Further studies are needed regarding these two biomarkers in relation to temperature changes.

On the other hand, a marked increase in CAT activity and LPO was observed at higher temperatures, particularly in combination with increasing bioplastic concentrations. Similarly, Na et al. [38] reported that in *D. magna* exposed to PE fragments, elevated temperature significantly enhanced acute sublethal toxicity, as evidenced by increased LPO and total antioxidant capacity. Also, Han et al. [35] observed a non-significant increase in CAT activity in *A. franciscana* exposed to PS MPs (0.2 mg L^−1^ and 2 mg L^−1^) for 14 days at 30 °C. They observed, however, an increase in acid phosphatase (ACP) activity, which suggests that PS and temperature have combined adverse effects on *Artemia* and induce oxidative stress. In contrast, Beltrán-de la Torre et al. [89] assessed the combined effects of acute exposure to PE microspheres and increasing temperatures in the fiddler crab *Minuca rapax* and found increased SOD and CAT activities along with decreased LPO. However, these changes in antioxidant system profiles were driven solely by temperature, with no significant effects of PE microspheres or their interaction with temperature. SOD and CAT probably contend for free radicals, avoiding oxidative damage. The results obtained in *A. franciscana* exposed to different PHBV concentrations and different temperatures are not easily interpreted. This may reflect the limited number of studies addressing how temperature and MP exposure modulate biochemical responses in crustaceans, which hinders the identification of consistent patterns or underlying mechanisms.

In the present study, although a combined effect of temperature and PHBV concentration was detected, LPO was higher in CTRL organisms at elevated temperatures, suggesting that temperature alone may induce oxidative damage. Na et al. [38] also observed that temperature alone increased LPO without affecting total antioxidant capacity in *D. magna*. A comparable pattern was observed in *Artemia*, where LPO levels were higher at 29 °C than at 25 °C in the CTRL group, without significant changes in CAT activity. However, GST activity was higher in the CTRL group at 29 °C than at 25 °C. Although MDA levels decreased after exposure to PHBV at 29 °C, they were always higher than in the experiment conducted at 25 °C. Previous studies have shown that exposure to conventional MPs, such as PS, alter both the content and profile of fatty acids in *D. magna*, reflecting disruptions in lipid metabolism and energy allocation [90]. These results suggest that PHBV may relax the need for fluidity-driven compensation, contrasting with conventional MPs, which are typically associated with increased oxidative stress and membrane destabilization [91].

The present study provides further evidence that exposure to PHBV bioplastics elicits physiological responses in *A. franciscana* that differ from those typically reported for conventional MPs. When fatty acid composition is integrated with growth performance, oxidative status and digestive alterations, the results support the hypothesis that PHBV exposure is associated with organismal-level physiological responses that may extend beyond purely physical interactions. Fatty acid profiles were primarily structured by exposure time (7, 14 and 21 days) and temperature (25 and 29 °C), indicating progressive lipid remodeling and thermal modulation of membrane composition. Superimposed on these general patterns, exposure to the highest PHBV concentration was associated with more subtle and less consistent changes, including variation in selected long-chain PUFAs. Consistent with previous findings [41], PHBV exposure is associated with reduced LPO, suggesting that lipid remodeling occurs in the absence of a classical oxidative stress response. Together, these results indicate that PHBV exposure promotes time- and temperature-dependent physiological adjustments that are distinct from those typically observed for conventional MPs while highlighting that treatment-related effects on fatty acid composition are comparatively moderate and require cautious interpretation. These findings underscore the importance of considering bioplastics separately in ecological risk assessments.

It should be noted that the PHBV concentrations employed in this study (25 and 100 mg·L^−1^) exceed current environmental estimates of MP contamination in marine ecosystems. Although such high-exposure designs are common in ecotoxicological research to detect mechanistic and sublethal responses [92], extrapolation of these findings to natural ecosystems should be made with caution.

## 5. Conclusions

This study demonstrates that exposure to high concentrations of PHBV MPs, alone and in combination with increased temperature, induces measurable physiological and biochemical responses in *A. franciscana*. Growth and development increased in the presence of PHBV, suggesting *Artemia*’s ability to adapt to bioplastic exposure or a potential ability to derive energy from PHBV particles. Although significant changes were observed in antioxidant and esterase activities, as well as in fatty acid composition, these responses did not translate into oxidative damage, suggesting the activation of effective compensatory mechanisms. Lipid remodeling was primarily driven by time and temperature, while higher PHBV concentrations were associated with more subtle changes in specific long-chain PUFAs.

On the other hand, PHBV caused adverse impacts on intestinal tissues that were enhanced by temperature, although these changes suggest a limited physiological disruption compared to conventional MPs. In addition, changes in PHBV particle length distributions after gut passage suggest that *Artemia* may contribute to the transformation and redistribution of secondary MPs in aquatic systems.

Taken together, the results suggest that PHBV affects organismal metabolism beyond purely physical or toxic effects. Overall, these findings highlight that the effects of micro-bioplastics such as PHBV may differ from those typically reported for conventional MPs and emphasize the importance of considering multiple environmental stressors and exposure durations when assessing their ecological risks in aquatic systems.

## Data Availability

The original data presented in this study are included in the article/Supplementary Materials; further inquiries can be directed to the corresponding author.

## Supplementary Materials

The following supporting information can be downloaded at https://www.mdpi.com/article/10.3390/toxics14060533/s1: Figure S1. Chemical structure of Poly(3-hydroxybutyrate-co-3-hydroxyvalerate) (PHBV). Figure S2: Histograms of PHBV particle length distributions at 0, 14, and 21 days, representing particles recovered from fecal pellets of individuals under different PHBV concentrations and temperature exposures; Table S1: Two-way ANOVA analysis of the effects of exposure time (ET: 7, 14, and 21 days) and concentration (C: CTRL, PHBV25 and PHBV100); Table S2: Two-way ANOVA analysis of the effects of temperature (T: 25 °C and 29 °C) and concentration (C: CTRL, PHBV25 and PHBV100).
