# Supplementary material for: Time- and Temperature-Dependent Effects of PHBV on Physiological Responses in Brine Shrimp
Source: Toxics. 2026 Jun 20;14(6):533. doi: 10.3390/toxics14060533 (PMC13307604; doi:10.3390/toxics14060533)
Supplement: Supplementary file 1 [file toxics-14-00533-s001.zip › toxics-4339322-supplementary.pdf]

Time- and Temperature-Dependent Effects of PHBV on Physiological Responses in Brine Shrimp

Natalia S. Buzzi [1,2,*], Anna Jáuregui [3], Anna Marín [3], Juan C. Navarro [4], Mar Llorca [4], Myriam Lizanda [4], María Constanza Díaz Andrade [2,5,6], Ana Carolina Moya [2,5,6], José Gámez-Pérez [3], Luis Cabedo [3] and Inmaculada Varó [4,*]

[1] Instituto Argentino de Oceanografía (IADO-CONICET/UNS), CCT-Bahía Blanca, Camino La Carrindanga, km 7.5, Edificio E1, Bahía Blanca B8000FWB, Buenos Aires, Argentina

[2] Departamento de Biología, Bioquímica y Farmacia, Universidad Nacional del Sur (UNS), San Juan 671, Bahía Blanca B8000ICN, Buenos Aires, Argentina; mcandrade@criba.edu.ar (M.C.D.A.); acmoya83@gmail.com (A.C.M.)

[3] Grupo de Polímeros y Materiales Avanzados (PIMA), Universitat Jaume I, Avinguda de Vicent Sos Baynat, 12006 Castelló de la Plana, Catellón, Spain; ajauregu@uji.es (A.J.); anmarin@uji.es (A.M.); gamez@uji.es (J.G.-P.); lcabedo@uji.es (L.C.)

[4] Instituto de Acuicultura Torre de la Sal (IATS, CSIC), 12595 Ribera de Cabanes, Castellón, Spain; jc.navarro@csic.es (J.C.N.); mllorcallovet@gmail.com (M.L.); myriamlizandapiqueras@gmail.com (M.L.)

[5] Instituto de Ciencias Biológicas y Biomédicas del Sur (INBIOSUR), Universidad Nacional del Sur (UNS)-CONICET, San Juan 671, Bahía Blanca B8000ICN, Buenos Aires, Argentina

[6] Grupo de Investigación en Morfología e Histología Animal Comparada (GIMHAC) (INBIOSUR-UNS-CONICET), Bahía Blanca B8000ICN, Buenos Aires, Argentina

* Correspondence: nbuzzi@criba.edu.ar (N.S.B.); inma@iats.csic.es (I.V.)

SUPPLEMENTARY MATERIAL

Table S1. Two-way ANOVA analysis on the effects of Exposure Time (ET: 7, 14, and 21 days) and Concentration (C: CTRL, PHBV25 and PHBV100).

Parameter	df	ET	C	ET x C
Body length	MS	218.62	23.90	2.69
	F	207.39	22.67	2.55
	P	< 0.001	< 0.001	0.04
	η^2_p	0.664	0.178	0.46
CAT	MS	2216.19	1236.82	223.80
	F	51.28	28.62	5.18
	P	< 0.001	< 0.001	0.004
	η^2_p	0.823	0.722	0.485
GST	MS	412.13	966.67	1050.11
	F	3.33	7.80	8.47
	P	0.056	0.003	< 0.001
	η^2_p	0.241	0.426	0.617

ChE	MS	11.27	4.38	1.17
	F	45.53	17.69	4.74
	P	< 0.001	< 0.001	0.011
	η^2_p	0.859	0.702	0.558
CbE	MS	759.10	18.38	18.18
	F	144.25	3.49	3.46
	P	< 0.001	0.049	0.026
	η^2_p	0.990	0.250	0.397
LPO	MS	7.57	11.92	2.57
	F	9.32	14.68	3.17
	P	0.001	< 0.001	0.036
	η^2_p	0.482	0.595	0.388
Particle length*	MS	0.222	0.73	2.93
	F	4.84	15.94	63.86
	P	0.008	< 0.0001	< 0.0001
	η^2_p	0.008	0.013	0.092

MS: mean square; *F*: sum of mean square of main effect/sum of mean square error; η^2_p: partial eta-squared. *Conditions differ: Exposure Time (ET: 0, 14, and 21 days) and Concentration (C: PHBV25 and PHBV100).

Table S2. Two-way ANOVA analysis on the effects of Temperature (T: 25 °C and 29 °C) and Concentration (C: CTRL, PHBV25 and PHBV100).

Parameter	df	T	C	T x C
Body length	MS	13.07	20.76	3.33
	F	10.96	17.41	2.80
	P	0.001	< 0.001	0.065
	η^2_p	0.073	0.199	0.038
CAT	MS	1472.89	228.63	845.68
	F	28.93	4.49	16.61
	P	< 0.001	0.022	< 0.001
	η^2_p	0.547	0.272	0.581
GST	MS	452.67	2310.31	999.04
	F	2.24	11.42	4.94
	P	0.148	< 0.001	0.016
	η^2_p	0.085	0.488	0.291
ChE	MS	4.75	0.03	0.041
	F	218.73	1.27	1.87
	P	< 0.001	0.302	0.179
	η^2_p	0.912	0.108	0.151
CbE	MS	5.08	0.04	0.03
	F	365.46	2.80	2.38

	P	< 0.001	0.081	0.115
	η^2_p	0.941	0.196	0.172
	MS	3.39	0.55	0.139
LPO	F	141.43	22.79	5.80
	P	< 0.001	< 0.001	0.009
	η^2_p	0.860	0.665	0.335
	MS	0.243	3.733	1.702
Particle length*	F	4.55	70.11	31.95
	P	0.038	<0.001	<0.001
	η^2_p	0.005	0.077	0.037

MS: mean square; *F*: sum of mean square of main effect/sum of mean square error; η^2_p: partial eta-squared. *Conditions differ: Temperature (T: 25 °C and 29 °C) and Concentration (C: PHBV25 and PHBV100).

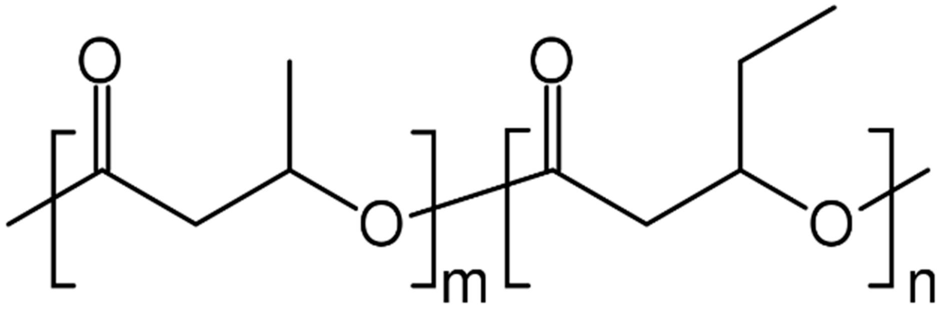

Figure S1. Chemical structure of poly(3-hydroxybutyrate-co-3-hydroxyvalerate) (PHBV).

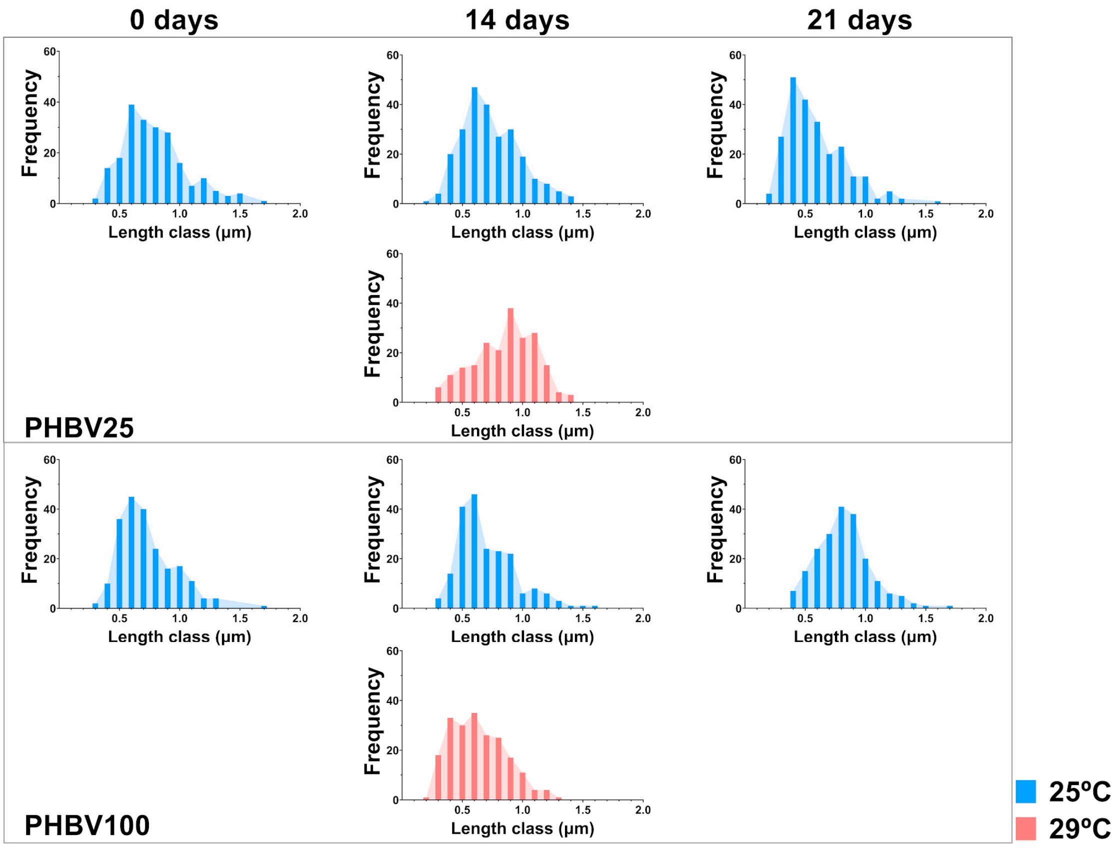

Figure S2. Histograms of PHBV particle length distributions at 0, 14, and 21 days, representing particles recovered from fecal pellets of individuals under different PHBV concentrations and temperatures exposure.
